# Augmentation of hypoxia-inducible factor-1-alpha in reinfused blood cells enhances diabetic ischemic wound closure in mice

**DOI:** 10.18632/oncotarget.23214

**Published:** 2017-12-13

**Authors:** Huan Wang, Yufeng Feng, Xiaoju Jin, Rong Xia, Yong Cheng, Xiaoqian Liu, Nana Zhu, Xun Zhou, Lei Yin, Jianrong Guo

**Affiliations:** ^1^ Department of Anesthesiology, Gongli Hospital, The Second Military Medical University, Shanghai 200135, China; ^2^ Department of Anesthesiology,The First Affiliated Hospital of Xiamen University, Xiamen 361003, China; ^3^ Department of Anesthesiology, Yijishan Hospital Affiliated to Wannan Medical College, Wuhu 241001, China; ^4^ Transfusion Department, Huashan Hospital, Fudan University, Shanghai 200040, China

**Keywords:** diabetic wound closure, blood re-infusion, hypoxia-inducible factor-1a (HIF-1a), vascular endothelial growth factor (VEGF)

## Abstract

Diabetes-associated dysfunction in angiogenesis predominantly contributes to impairment of wound closure, but a role of hypoxia-inducible factor 1 alpha (HIF-1a) in the process remain poorly understood. Here, we examined whether expression of HIF-1a in re-infused blood cells may improve the diabetic wound closure in mice. We found that that expression of HIF-1a in re-infused isogeneic blood cells significantly improved diabetic wound healing in mice, seemingly through augmentation of wound-associated angiogenesis. Mechanistically, expression of HIF-1a in re-infused blood cells significantly increased macrophage infiltration at the wound site, and macrophages produced vascular endothelial growth factor A (VEGF-A) to promote wound-associated angiogenesis. Together, our data suggest that augmentation of HIF-1a in reinfused blood cells may enhance diabetic ischemic wound closure.

## INTRODUCTION

Diabetes, which affects billions of people worldwide, is a prevalent metabolic disease characterized with increased blood sugar [1]. In diabetics, wounds tend to heal much more slowly than normal, largely due to high blood glucose levels, poor circulation, diabetic neuropathy, dysfunction and deficiency in immune system and infection on the wound [1]. Mechanistically, Diabetes impairs numerous components of wound healing, resulting in narrowing of blood vessels, stiffening of the arteries, and dysfunction of red blood cells and leukocytes, all contributing to delay of wound closure due to reduced oxygen and nutrient supply, which are required for completing healing process by proper angiogenic homeostasis, infiltration of inflammatory cells, macrophage differentiation, extracellular matrix deposition and fibroblast transformation. These impairments affect a wide variety of tissues including myocardium, skeletal muscle, neural system and skin [2].

Although dealing with diabetic wound closure requires treating multiple defects in diabetes, the most critical approach has been shown to be adequately control of blood sugar and enhancement of revascularization on wound through augmentation of pro-angiogenic factors, like vascular endothelial growth factor-A (VEGF-A) [3]. VEGF-A is a member of VEGF family with essential roles in vascular and lymphatic growth and patterning [4]. VEGF-A acts through at least two receptors (Flt-1 and Flk-1), expressed primarily on endothelial cells, for vasculature induction and maintenance [5–7]. VEGF facilitates tissue repair through augmentation of vascular permeability, promotion of inflammatory cell infiltration, and enhancement of the migration and proliferation of endothelial cells [4]. Interestingly, VEGF-A has been shown to play a pivotal role in promoting diabetic wound closure in diabetic mice [8–10].

Hypoxia-inducible factor 1 alpha (HIF-1a) is the regulatory subunit of a master regulator of hypoxia, HIF-1 [11–13]. Specifically, HIF-1a enhances VEGF-A expression through directly binding to VEGF promoter [14]. However, the protective mechanism of HIF-1a in diabetic wound closure has not been systemically examined.

Macrophages are a type of leukocytes responsible for engulfing and digesting cellular debris, foreign substances and abnormal cells by phagocytosis [15]. Recently, besides these traditionally defined functions, some macrophages were shown to express high levels of chemokines, enzymes and growth factors (including VEGF-A), which are associated with wound healing or tissue-remodeling [16–21].

Autologous blood transfusion is a safe and effective clinical procedure, with advantages of absence of transfusion of allogeneic blood derivatives, and lack of adverse effects such as infections, febrile reactions, hemolysis and transmission of viral diseases [22]. Self-transfusion or blood re-infusion programs is applied in elective surgery and are gradually used in other areas [23]. Here, we examined whether expression of HIF-1a in re-infused blood cells may improve the diabetic wound closure in mice.

## RESULTS

### Expression of HIF-1a in isolated blood cells

**Figure 1:**
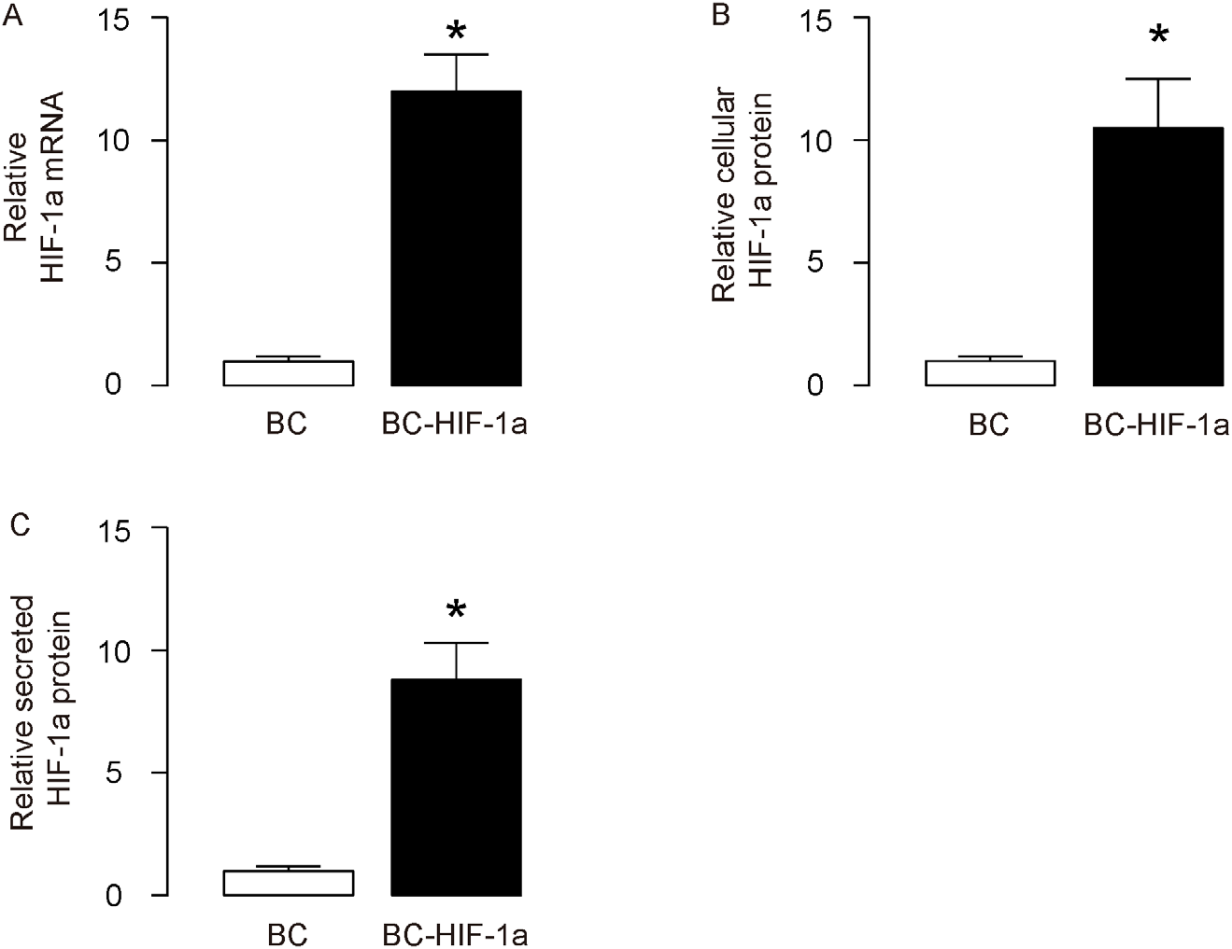
Expression of HIF-1a in isolated blood cells Mouse blood cells (BCs) were isolated and then transduced with lentivirus carrying either null (as a control) or recombinant HIF-1a under a CMV promoter. **(A)** HIF-1a mRNA levels in the transduced BCs (BC versus BC-HIF-1a) were determined by RT-qPCR. **(B)** Cellular HIF-1a protein levels in the transduced BCs were determined by ELISA. **(C)** Secreted HIF-1a protein levels in the transduced BCs were determined by ELISA. ^*^p<0.05. N=5.

In order to evaluate the effects of HIF-1a on diabetic wound closure, we isolated mouse blood cells (BCs) and transduced them with lentivirus carrying either null (as a control) or recombinant HIF-1a under a CMV promoter. The HIF-1a mRNA levels in the transduced BCs were determined by RT-qPCR (Figure 1A), and the cellular and secreted HIF-1a protein levels in the transduced BCs were determined by ELISA (Figure 1B-1C), respectively. These data confirmed the increases in HIF-1a levels in the transduced BCs.

### Diabetic wound closure model

**Figure 2:**
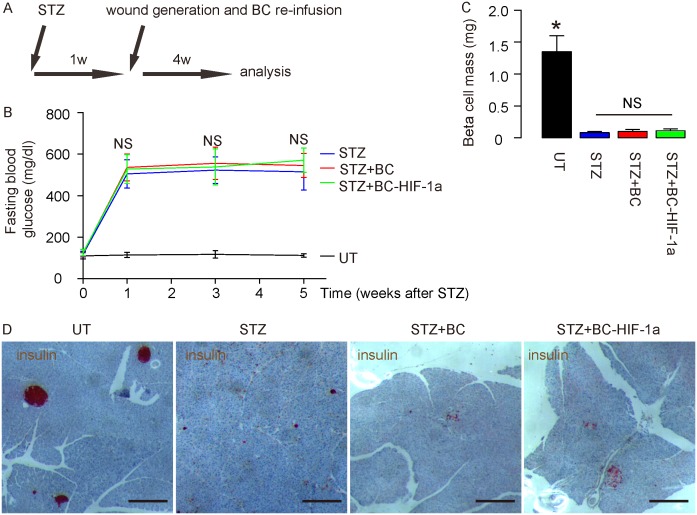
Diabetic wound closure model **(A)** The mice were randomly divided into 4 groups of 10 each: Group 1, mice received i.p. injection of saline of same dose of STZ. One week later, wound was generated (UT). Group 2, mice received i.p. injection of STZ. One week later, wound was generated (STZ). Group 3: mice received i.p. injection of STZ. One week later, wound was generated and mice received i.v. re-infusion of 10^7^ donor null -transduced BCs (STZ+BCs). Group 4: mice received i.p. injection of STZ. One week later, wound was generated and mice received i.v. re-infusion of 10^7^ donor HIF-1a-transduced BCs (STZ+BC-HIF-1a). After wound was generated, all the mice were followed for 4 weeks and the wounds were monitored. **(B)** Fasting blood glucose levels. **(C)** Quantification of beta cell mass. **(D)** Representative insulin staining images on pancreatic sections. ^*^p<0.05. NS: non-significant. N=10. Scale bars are 200μm.

The therapeutic effects of re-infusion of HIF-1a-expressing blood cells on diabetic wound closure were then evaluated. The mice were randomly divided into 4 groups of 10 each (The number of the mice per group was decided by a power calculation): Group 1, mice received i.p. injection of saline of same dose of STZ. One week later, wound was generated (UT). Group 2, mice received i.p. injection of STZ. One week later, wound was generated (STZ). Group 3: mice received i.p. injection of STZ. One week later, wound was generated and mice received i.v. re-infusion of 10^7^ donor null -transduced BCs (STZ+BCs). Group 4: mice received i.p. injection of STZ. One week later, wound was generated and mice received i.v. re-infusion of 10^7^ donor HIF-1a-transduced BCs (STZ+BC-HIF-1a). After wound was generated, all the mice were followed for 4 weeks and the wounds were monitored (Figure 2A). Neither wound formation nor re-infusion of BCs altered the diabetic state, in terms of fasting blood glucose levels (Figure 2B), and beta cell mass, shown by quantification (Figure 2C) and by representative insulin staining images on pancreatic sections (Figure 2D).

### Expression of HIF-1a in re-infused BCs improves diabetic wound closure

**Figure 3:**
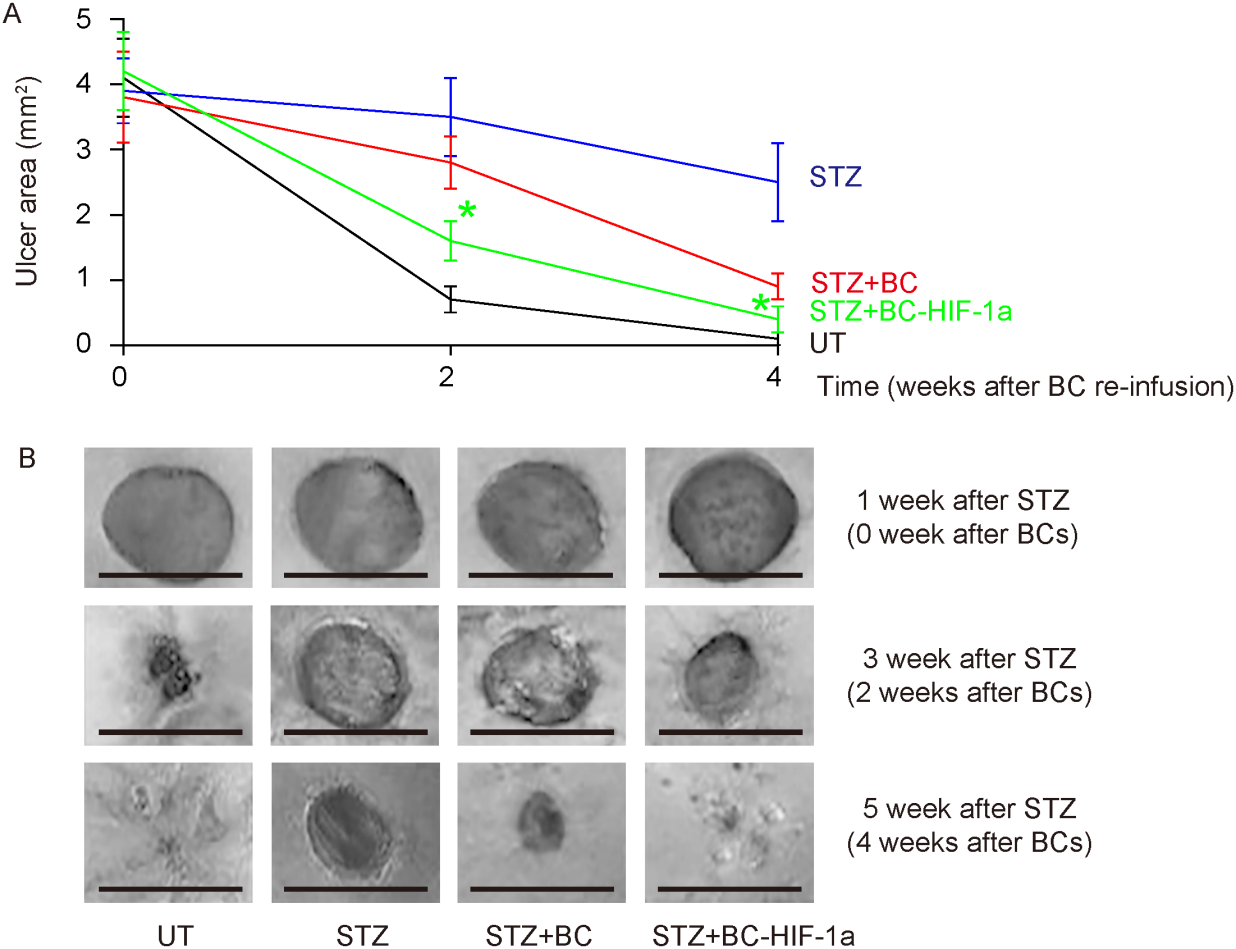
Expression of HIF-1a in re-infused BCs improves diabetic wound closure The wound closure was followed for 4 weeks. **(A)** The wound area was quantified at 1, 3, 5 weeks after STZ (0, 2, 4 weeks after wound generation). **(B)** Representative wound images. ^*^p<0.05 (STZ+BC versus STZ+BC-HIF-1a). N=10. Scale bars are 2mm.

Next, we examined the effects of HIF-1a expression in re-infused BCs on diabetic wound closure. We found that the wounds were completely closed in 4 weeks in UT mice (no STZ, no BCs; Figure 3A-3B). The wounds only slightly closed in STZ mice (STZ, no BCs, Figure 3A-3B). Re-infusion of BCs improved wound closure, but re-infusion of BC-HIF-1a (STZ, BC-HIF-1a) further improved wound closure, compared to re-infusion of BCs (STZ, BC) (Figure 3A-3B). Thus, expression of HIF-1a in re-infused BCs improves diabetic wound closure.

### Expression of HIF-1a in re-infused BCs enhances wound-associated angiogenesis

**Figure 4:**
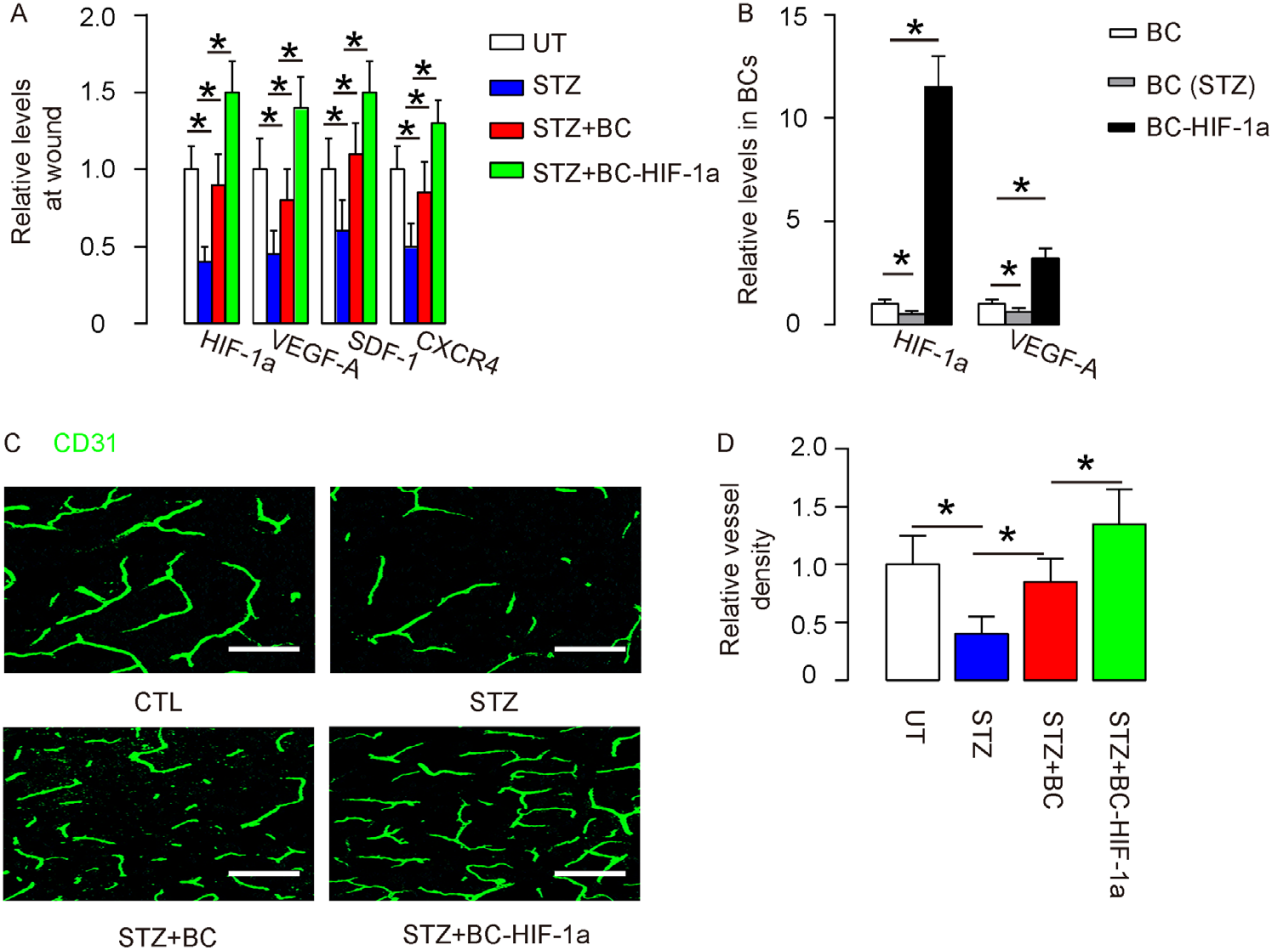
Expression of HIF-1a in re-infused BCs enhances wound-associated angiogenesis **(A)** RT-qPCR for HIF-1a, VEGF-A, SDF-1 and CXCR4 on the wound site. **(B)** RT-qPCR for HIF-1a and VEGF-A in BCs from control, STZ-treated mice and in BC-HIF-1a. **(C-D)** Vessel density was measured in mice 4 weeks after wound generation/BC re-infusion. (C) Representative CD31 staining on the site of the wound. (D) Quantification of vessel density on the site of the wound. ^*^p<0.05. N=10. Scale bars are 50μm.

First, the values of HIF-1a, VEGF-A, stromal cell-derived factor 1 (SDF-1) and CXCR4 on the wound site were analyzed. The results showed that all these factors decreased at the wound site by STZ, but were attenuated by BCs and further increased by re-infusion of BC-HIF-1a (Figure 4A). Interestingly, the values of HIF-1a and VEGF-A were found to downregulate in BCs from STZ-treated mice, but both significantly increased in BC-HIF-1a (Figure 4B). Vessel density was then measured in mice 4 weeks after wound generation/BC re-infusion. We found that the vessel density was significantly reduced in STZ-treated mice, compared to control UT mice (Figure 4C-4D). Re-infusion of BCs increased vessel density on wounds, but re-infusion of BC-HIF-1a increased greater vessel density on wounds, compared to re-infusion of BCs (Figure 4C-4D). Thus, expression of HIF-1a in re-infused BCs enhances wound-associated angiogenesis.

### Expression of HIF-1a in re-infused BCs enhances wound-associated angiogenesis through increasing trophic macrophages

**Figure 5:**
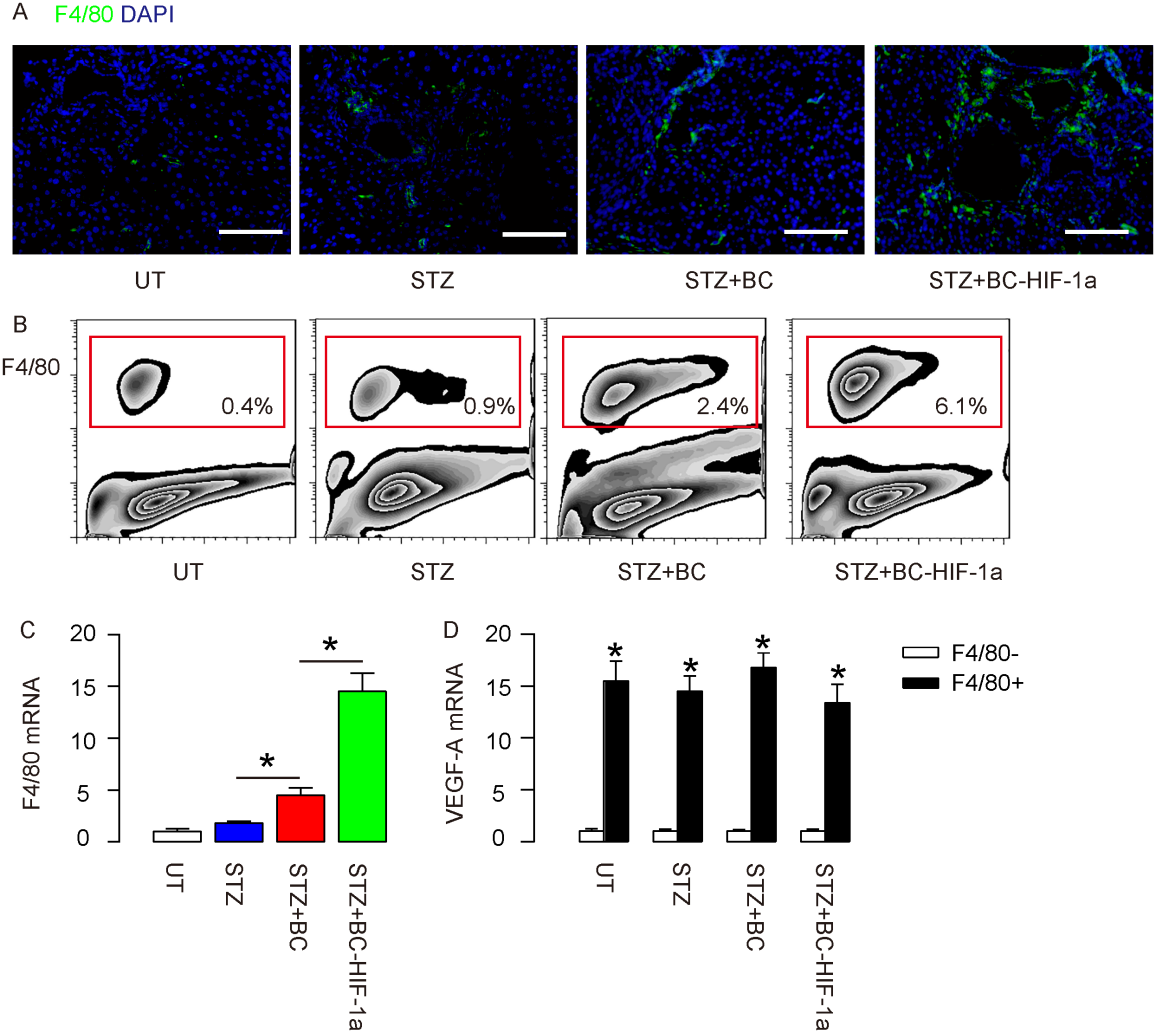
Expression of HIF-1a in re-infused BCs enhances wound-associated angiogenesis through increasing trophic macrophages **(A)** Representative F4/80 staining on the site of the wound. **(B)** Representative flow charts for analysis and sorting of F4/80+ macrophages at the site of the wound in mice. **(C)** RT-qPCR for F4/80 in the wound tissue. **(D)** RT-qPCR for VEGF-A in the sorted F4/80+ cells versus F4/80- cells from the wound tissue of the mice. ^*^p<0.05. N=10. Scale bars are 50μm.

We examined the tissue sections on the site of wound and to our surprise, we detected significantly higher number of F4/80+ macrophages in mice that received re-infusion of BCs, compared to UT and STZ-mice (Figure 5A). Moreover, the number of F4/80+ macrophages in mice that received re-infusion of BC-HIF-1a was even higher than those that received re-infusion of BCs (Figure 5A). We digested the wound tissue and analyzed the percentage of F4/80+ cells by flow cytometry, and the data confirmed our finding by immunohistochemistry (Figure 5B). These data were further confirmed by RT-qPCR for F4/80 in the wound tissue (Figure 5C). Finally, we sorted F4/80+ cells versus F4/80- cells from the wound tissue of the mice, and we found that F4/80+ cells expressed significantly higher levels of VEGF-A (Figure 5D). These data suggest that the macrophages at the site of the wound may be the major source of pro-angiogenic factor VEGF-A, and contribute predominantly to the improved angiogenesis and diabetic wound closure by re-infusion of HIF-1a-expressing BCs.

## DISCUSSION

A number of factors contribute to wound closure deficiencies in diabetes, including reduced or impaired growth factor production, inferior angiogenic response, macrophage dysfunction, improper collagen accumulation, damages of epidermal barrier, disorder in keratinocyte and fibroblast migration and proliferation, and imbalance between the accumulation of extracellular matrix and their degradation [2].

VEGF-A is one of the most important growth factors involved in the regulation of the wound healing. Numerous evident suggests that diabetic wound closure disorder may be largely resulting from insufficient VEGF-A production and release at the wound site [10, 24, 25]. Blood cell re-infusion is supposed to provide an additional VEGF-A source to augment the angiogenesis process during wound closure, since these re-infused cells are supposed to be devoid of diabetes-associated dysfunction [2]. Nevertheless, in the current study, we showed compelling evidence that the majority of the increased VEGF-A appeared to be from recruited macrophages at the wound site. Of note, the expression of HIF-1a in the re-infused BCs significantly increased macrophage infiltration. Hence, the re-infused BCs themselves seemed to have greater potential in attracting macrophages to the wound sites, possibly due to the intact and healthy status of these cells compared to the BCs that had been exposed to sustained hyperglycemia in the diabetes animals [2]. On the other hand, HIF-1a improved the potential of BCs in attracting macrophages. Previous studies have shown that HIF-1a regulates VEGF signaling [26–28], which subsequently activates SDF-1/CXCR4 regulatory axis that are critical for macrophage recruitment and differentiation [19]. Of note, here we found that overexpression of HIF-1a in BCs significantly increased levels of HIF-1a, VEGF-A, SDF-1 and CXCR4 at the wound site, suggesting involvement of these mechanisms.

Together, our study demonstrates a potential beneficial effect of overexpression of HIF-1a in BCs before self-transfusion, suggesting that genetic modulation of BCs may improve their therapeutic outcome in disease treatment. Indeed, various molecular technologies have created novel systems for sustained topical delivery as a major advance in tissue engineering. Novel discoveries of disease molecular pathogenesis and of molecular targeting hold the promise of creating innovative therapeutic approaches for human diseases, including diabetic wound closure. One of the most critical remaining steps is the integration of these resources to make the technology developed at the bench available to patients at the bedside.

## MATERIALS AND METHODS

### Protocol approval

All the experimental procedures were approved by the research committee at Gongli Hospital, the Second Military Medical University. All animal experiments were approved by the Institutional Animal Care and Use Committee at Gongli Hospital, the Second Military Medical University. Surgeries were performed in accordance with the Principles of Laboratory Care, supervised by a qualified veterinarian.

### Isolation, transduction and infusion of isogeneic blood cells

Mouse hematopoietic cells were isolated from isogeneic mice, as described [29]. Mouse blood cells were cultured in Dulbecco’s Modified Eagle’s Medium (DMEM) supplemented with 20% fetal bovine serum (Invitrogen, Carlsbad, CA, USA) in a humidified chamber with 5% CO_2_ at 37 °C. The isolated blood cells were transduced with lentivirus carrying either null (as a control) or recombinant HIF-1a under a CMV promoter (Origene, Shanghai, China). For blood cell self-transfusion, 10^7^ donor null/HIF-1a-transduced blood cells were re-infused into the circulation of receipt mice via tail vein at the same time of induction of skin wound.

### Macrophage analysis by flow cytometry

For analysis and purification of macrophages, the targeted tissue was digested by 0.25% Trypsin (Becton-Dickinson Biosciences, San Jose, CA, USA) into single cell preparation, and re-suspended at a density of 10^6^ cells/ml in PBS. Afterwards, the cell preparation was incubated with PE-conjugated F4/80 antibody or isotype control for 15 minutes, before subjected to flow cytometry analysis and sorting.

### Diabetic mice and blood cell transplantation

Diabetes was induced in 12-week-old male C57/BL6 mice (SLAC Laboratory Animal, Shanghai, China) by intraperitoneal injection of streptozotocin (STZ) at a dose of 150 mg/kg body weight. One week later, the mice developed sustained high blood glucose. Then, a 2mm-diameter squire skin wound was generated in these mice, using forceps and scissor at dorsal side without injuring the underlying muscle. The wounds were covered with occlusive dressing to prevent infection. The wounds were photographed at 0, 2 and 4 weeks post injury. The wounds were monitored and wounded area was determined using NIH imageJ software.

### Quantitative real-time PCR (RT-qPCR)

Total RNA were extracted using the RNeasy mini kit (Qiagen, Hilden, Germany). Complementary DNA preparation and quantitative real-time PCR (RT-qPCR) were routinely done. All primers (HIF-a: QT01039542; VEGF-A: QT00160769; F4/80: QT00099617; GAPDH: QT01658692; SDF-1: QT00161112; CXCR4: QT00249305) were purchased from Qiagen. Data were collected and analyzed using 2-ΔΔCt method. Values of genes were first normalized against GAPDH, and then compared to experimental controls.

### ELISA

Total protein was isolated and HIF-1a levels were determined by an ELISA kit (DYC1935-2; R&D System, Los Angeles, CA, USA).

### Histology and immunostaining

Mouse pancreas or targeted skin tissue was dissected out and fixed in 4% paraformaldehyde (PFA, Sigma-Aldrich) for 8 hours. After overnight incubation in 30% sucrose, samples were frozen in liquid nitrogen and embedded in tissue freezing medium. Immunostaining has been done. Primary antibodies were rat anti-F4/80 (MA5-16624; 1:100; Invitrogen, Carlsbad, CA, USA), rat anti-CD31 (553370; 1:100; Becton-Dickinson Biosciences) and rat anti-insulin (AB7842; 1:300; Abcam, Carlsbad, CA, USA). DAPI (1:1000; 4,6-Diamidino-2-phenylindole) was used to stain nuclei.

### Fasting blood glucose, beta cell mass and vessel density

Fasting blood glucose levels were measured using Accu-119 Chek glucose meter (Roche, Nutley, NJ, USA). Beta cell mass measurement has done using generally accepted method [30]. Briefly, mouse pancreas was weighed, fixed, cryo-protected in 30% sucrose overnight before freezing. Sections at 100μm intervals from whole pancreas were immunostained for insulin and analyzed using Image J software. The relative area of insulin+ cells was determined by the ratio of area occupied by insulin+ cells versus area of total pancreatic tissue. The beta cell mass was calculated as the product of the relative insulin+ cell area and the weight of the pancreas. The vessel density was determined as the ratio of CD31+ area versus total area.

### Statistical analysis

All values represent the mean ± standard deviation (SD). Statistical analysis of group differences was carried out using a one-way analysis of variance (ANOVA) test followed by the Fisher’s Exact Test to compare two groups (GraphPad Software, Inc. La Jolla, CA, USA). A value of p<0.05 was considered statistically significant after Bonferroni correction.
